# Determinants of GBP Recruitment to *Toxoplasma gondii* Vacuoles and the Parasitic Factors That Control It

**DOI:** 10.1371/journal.pone.0024434

**Published:** 2011-09-08

**Authors:** Sebastian Virreira Winter, Wendy Niedelman, Kirk D. Jensen, Emily E. Rosowski, Lindsay Julien, Eric Spooner, Kacey Caradonna, Barbara A. Burleigh, Jeroen P. J. Saeij, Hidde L. Ploegh, Eva-Maria Frickel

**Affiliations:** 1 Whitehead Institute for Biomedical Research, Cambridge, Massachusetts, United States of America; 2 Department of Biology, Massachusetts Institute of Technology, Cambridge, Massachusetts, United States of America; 3 Department of Immunology and Infectious Diseases, Harvard School of Public Health, Boston, Massachusetts, United States of America; Univ. Georgia, United States of America

## Abstract

IFN-γ is a major cytokine that mediates resistance against the intracellular parasite *Toxoplasma gondii*. The p65 guanylate-binding proteins (GBPs) are strongly induced by IFN-γ. We studied the behavior of murine GBP1 (mGBP1) upon infection with *T. gondii in vitro* and confirmed that IFN-γ-dependent re-localization of mGBP1 to the parasitophorous vacuole (PV) correlates with the virulence type of the parasite. We identified three parasitic factors, ROP16, ROP18, and GRA15 that determine strain-specific accumulation of mGBP1 on the PV. These highly polymorphic proteins are held responsible for a large part of the strain-specific differences in virulence. Therefore, our data suggest that virulence of *T. gondii* in animals may rely in part on recognition by GBPs. However, phagosomes or vacuoles containing *Trypanosoma cruzi* did not recruit mGBP1. Co-immunoprecipitation revealed mGBP2, mGBP4, and mGBP5 as binding partners of mGBP1. Indeed, mGBP2 and mGBP5 co-localize with mGBP1 in *T. gondii-*infected cells. *T. gondii* thus elicits a cell-autonomous immune response in mice with GBPs involved. Three parasitic virulence factors and unknown IFN-γ-dependent host factors regulate this complex process. Depending on the virulence of the strains involved, numerous GBPs are brought to the PV as part of a large, multimeric structure to combat *T. gondii*.

## Introduction

Type II interferon or IFN-γ is essential to control survival and proliferation of intracellular pathogens and is the most important regulatory cytokine that combats infection with *Toxoplasma gondii*. IFN-γ upregulates the expression of hundreds of genes [1], [2], some of the most abundant of which are four families of GTPases: Mx proteins, very large inducible GTPases (VLIG), p47 immunity-related GTPases (IRGs), and p65 guanylate-binding proteins (GBPs). A common feature of the IFN-γ-inducible GTPases may be the mediation of innate resistance to many intracellular pathogens [3]. For instance, Mx proteins confer resistance to influenza virus [4]. Additionally, mice that lack certain p47 IRG genes show increased susceptibility to various bacteria and protozoa [5], [6]. Family members of the IRGs localize to the parasitophorous vacuole (PV) of *T. gondii* in IFN-γ-induced cells *in vitro* and mediate killing of the parasite by disruption of the PV [7]–[9]. However, virulent type I strains can evade vacuolar disruption and some IRGs are localized only to vacuoles that contain the less virulent type II or type III but not type I parasites [10], [11].

Toxoplasma secretes proteins from three secretory organelles, the micronemes, the rhoptries and the dense granules, into the host cell upon invasion [12], [13]. Some of these secreted proteins traffic back to the PV membrane (PVM) and it is likely that is where they exert their function [14]. Indeed, recent studies reported that the rhoptry protein kinase ROP18 of type I strains can phosphorylate the nucleotide binding site of the switch loop 1 of certain IRGs, which prevents subunit oligomerization and GTP hydrolysis, which in turn inhibits accumulation on the PVM and protects the parasite from being destroyed [15], [16].

Whereas the mouse genome has 23 copies of p47 GTPases, the human genome contains only two, both of which lack any IFN-γ response elements [17]. In contrast, one other main IFN-γ-inducible GTPase family, the conserved p65 GBPs, is represented with 13 copies in the mouse genome and seven genes in the human genome [18]. Detailed structural and mechanistic information is available for some family members [19]–[21].

Most GBPs are believed to be isoprenylated, endowing them with the ability to associate with intracellular membranes [22], [23]. Some links to antiviral activity [24], [25], as well as an antiproliferative function in endothelial cells and fibroblasts have been suggested [26]–[28]. Various GBPs are induced upon infection with *T. gondii in vivo* and localize to the PV of type II and III *T. gondii* strains in infected cells *in vitro* [29]. Furthermore, hGBP1 potentiates the anti-chlamydia effects of IFN-γ [30], in support of an antimicrobial role. However, how virulent *T. gondii* can evade accumulation of the GBPs at its PV is unclear. How recruitment of the GBPs is regulated and whether other proteins are involved is also not known.

We studied the behavior of mGBP1 *in vitro* in the course of infection of murine cells with various strains of *T. gondii* that differ in their virulence in mice. We conclude that the recognition of a parasite vacuole by mGBP1 is dependent on interferon-inducible host factors, the nucleotide-bound state of the GTPase, multimerization of the GTPase, and on the strain of the parasite. We further determined that mGBP1 exerts its function in conjunction with other members of the GBP family, namely GBP2 and GBP5. Three *T. gondii*-derived proteins, rhoptry protein 16 (ROP16), ROP18, and dense granule antigen 15 (GRA15), are partly responsible for the strain-specific accumulation of mGBP1 at the PV. Our study posits the function of mGBP1 to be that of a cell-autonomous immunity factor that distinguishes between *T. gondii* of different virulence. Along with the IRGs, GBPs may be considered as key factors mediating resistance to *T. gondii*.

## Results

### Virulent *T. gondii* mostly evades recognition of its parasitophorous vacuole by mGBP1

GBPs differentially localize to PVs formed by varying strains of *T. gondii* [29], but the molecular mechanisms that underlie this specific targeting are unknown. We investigated the decoration of the PV harboring *T. gondii* parasites that differ in virulence with mGBP1. To explore the kinetics of recruitment of mGBP1 as well as the percentage of mGBP1-positive vacuoles that contain type I, II or III *T. gondii* parasites, we produced a rabbit polyclonal antiserum to recombinant mGBP1. We also generated MEFs that stably overexpress either N-terminally FLAG-tagged mGBP1 or eGFP-mGBP1. In uninfected cells, both variants co-localized with endogenous mGBP1 upon stimulation with IFN-γ and were localized to punctate structures in the cytoplasm (Fig. S1).

**Figure 1:**
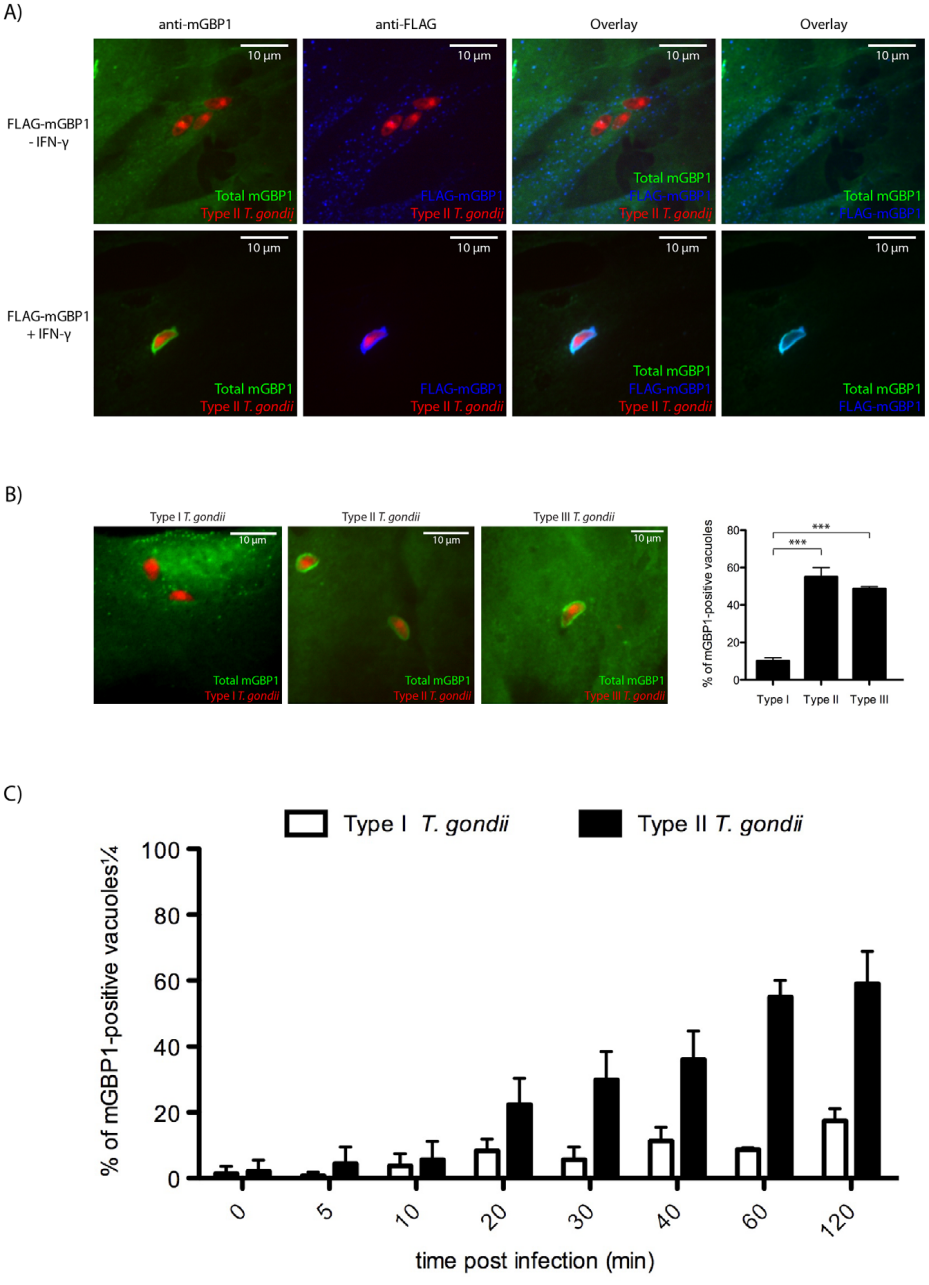
mGBP1 preferentially accumulates on type II (Pru) and III (CEP) but not on type I (RH) vacuoles. (A) MEFs that overexpress FLAG-mGBP1 were stimulated with 200 U/ml IFN-γ overnight and infected with mCherry-expressing type II *T. gondii* at an MOI between 5 and 10 for 1 h. (B) Representative pictures of IFN-γ-induced wild-type MEFs infected with either type I, II or III 1 h post-infection show that mGBP1 is preferentially recruited to nonvirulent types II and III vacuoles. The right panel shows the frequencies of mGBP1-positive vacuoles. However, when mGBP1 was recruited to the virulent type I (11% of the vacuoles), the intensity of recruitment was comparable to the recruitment to types II and III. The differences in extent of recruitment of mGBP1 to PVs between type I and types II and III are significant with *** p<0.001. Error bars represent standard deviations of three independent experiments. (C) The frequency of mGBP1-positive vacuoles of type I and type II *T. gondii* was determined at various time points. MEFs that overexpress FLAG-mGBP1 were induced with 200 U/ml IFN-γ overnight and infected with type I or type II *T. gondii* at an MOI between 5 and 10 before they were fixed after the indicated time. For each strain, at least 100 vacuoles of invaded *T. gondii* were checked for recruitment of mGBP1. Error bars represent standard deviations of three independent experiments. A rabbit polyclonal anti-mGBP1 antiserum and a mouse monoclonal anti-FLAG antibody (Sigma-Aldrich) were used for stainings. Pictures were taken with a spinning disk confocal microscope.

We observed recruitment of endogenous and FLAG-tagged mGBP1 to the vacuole of type II tachyzoites only when MEFs were pre-stimulated with IFN-γ (Fig. 1a), as reported [29]. This finding suggests the presence of either an IFN-γ-inducible co-factor or an IFN-γ-dependent modification of mGBP1 or its co-factors essential for vacuolar recognition by mGBP1. IFN-γ pretreated MEFs infected subsequently with *T. gondii* of different virulence types showed distinct patterns of recruitment 1 hour post-infection. We extended current studies to include type III (CEP) *T. gondii,* and while most type II (Pru) and type III (CEP) *T. gondii* showed ring-like accumulation of mGBP1 around the (PV), virulent type I (RH)-containing vacuoles are predominantly mGBP1-negative (Fig. 1b). Only 11% of type I vacuoles were decorated with mGBP1, as opposed to 55% of type II and 49% of type III vacuoles 1 hour post-infection (Fig. 1b).

We performed live cell imaging by confocal microscopy to track the extent and kinetics of eGFP-mGBP1 recruitment in MEFs. Unexpectedly, in some cases, virulent type I *T. gondii* escaped from vacuoles that had already recruited mGBP1 (Video S1, S2, S3). We never observed this phenomenon for vacuoles containing type II or III *T. gondii*. After type I escaped from vacuoles with an established, circular accumulation of mGBP1, two types of outcomes were observed: either the parasite stripped off mGBP1 and resided within the same cell without any new recruitment of mGBP1, or the parasite left the cell after escaping from the GTPase-decorated vacuole while the host cell died. Is mGBP1 recruitment to type I *T. gondii* rare and specific to a sub-fraction of these vacuoles, or do type I parasites rapidly strip off mGBP1 from the PV to escape immune detection? We analyzed the frequency of mGBP1-positive PVs for types I and II over time and recorded a noticeable difference of mGBP1-positive vacuoles only after approximately 20 min. At 2 hours post-infection, 63% of type II vacuoles are mGBP1-positive, versus only 20% of type I vacuoles (Fig. 1c). Taken together, we conclude that type I and II vacuoles are both generally capable to recruit mGBP1 and that this process takes place within approximately 30 minutes post-infection. Invasion in this assay is not synchronized and thus mGBP1 accumulates at a growing number of intracellular type II parasites. However, type I-containing vacuoles are able to strip off the GTPase at a timepoint 20 minutes or later post-infection. This process is fast and complete within a few minutes. Thus for type I parasites continuous recruitment to newly invaded parasites seems to be counteracted by active stripping and evasion strategies with the eventual result of only 20% of PVs being decorated by mGBP1 at 2 hours post-infection.

How recruitment is initiated, and whether or not the GBP-mediated response is specific for *T. gondii* or rather represents a more general reaction to intracellular microbes remain open questions. We therefore explored co-localization of mGBP1 with phagosomes and PVs of *Trypanosoma cruzi*. No recruitment of mGBP1 to phagocytosed zymosan or *T. cruzi* trypomastigotes was detectable 1 hour post-infection (Fig. S2 and Procedures S1). To determine if *T. cruzi* is not targeted by mGBP1 at all or if it can rapidly evade recruitment, localization of mGBP1 after infection was monitored over time. At no time did we detect targeting of mGBP1 to the PV of *T. cruzi* (data not shown).

### Virulence factors, including ROP16, ROP18 and GRA15, interfere with recruitment of mGBP1

**Figure 2:**
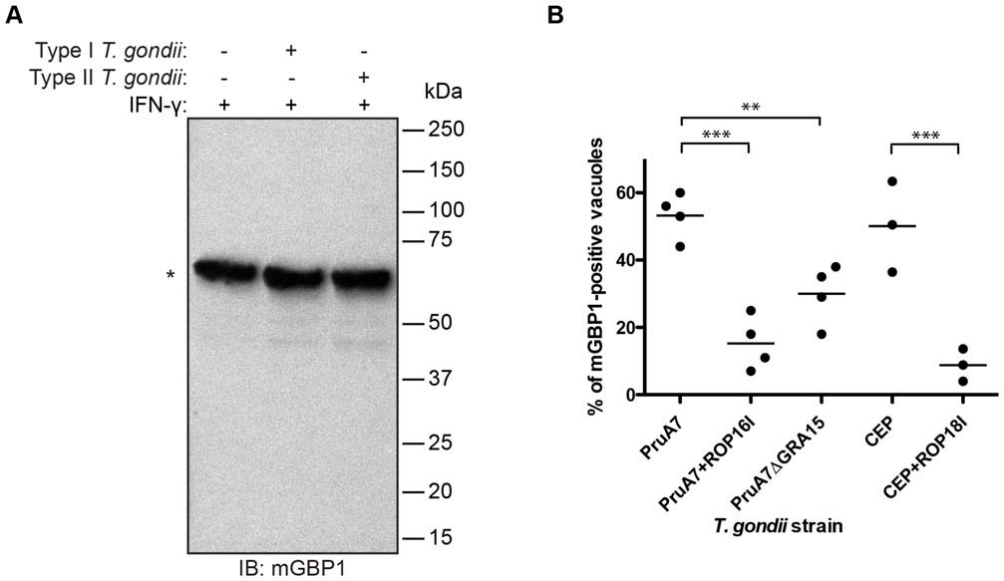
*T. gondii* cannot degrade mGBP1 but parasitic virulence factors interfere with recruitment of mGBP1. (A) Degradation of mGBP1 after invasion of *T. gondii* is not detectable by IB. RAW264.7 cells were induced with 200 U/ml IFN-γ overnight and infected with mCherry-expressing *T. gondii* (Pru or RH). Infected cells were separated from uninfected cells by FACS 2 h post-infection. For each lysate, 50 µg of protein, which was equivalent to 300,000 cells, were loaded per lane. The mGBP1 protein was detected by rabbit polyclonal anti-mGBP1 antiserum (*). (B) Type II strains with deleted GRA15, type II strains transgenic for the type I version of ROP16, and type III strains transgenic for the type I version of ROP18 show less mGBP1-positive vacuoles compared to the parental strain. MEFs that overexpress eGFP-mGBP1 were induced with 200 U/ml IFN-γ and infected with PruA7, a Pru type II *T. gondii* strain, PruA7 either lacking GRA15 or transgenic for the type I version of ROP16, CEP, a type II strain, or CEP transgenic for the type I version of ROP18. For each strain, at least 100 vacuoles of invaded *T. gondii* were checked for recruitment of mGBP1. Data represent four independent experiments. Decrease of mGBP1 recruitment is significant with ** p<0.01 and *** p<0.001.

What are underlying causes for type I *T. gondii’s* ability to evade mGBP1 recruitment and killing? We tested if virulent *T. gondii* could degrade mGBP1 upon invasion as a means to prevent recruitment of GBPs. IFN-γ-stimulated RAW264.7 cells were infected with fluorescent type I or type II *T. gondii*. Infected cells were separated from uninfected cells by FACS, and presence and levels of mGBP1 protein were analyzed by immunoblot (IB). No degradation of mGBP1 was observed by IB two hours post-infection in cells infected with either type I or type II *T. gondii* (Fig. 2a).

Next, we examined whether specific *T. gondii* factors mediate the difference in recruitment behavior of mGBP1 to the vacuole. We recently identified two *T. gondii* proteins that are delivered into the host cell cytosol upon invasion. These proteins activate host signaling pathways in a strain-specific manner: the rhoptry protein ROP16 is a tyrosine kinase, of which the type I and III copy can directly phosphorylate STAT3/6 [31], and the type II dense granule protein GRA15 activates the NFkB pathway [13]. Both play a role in the strain-specific differences in virulence. We therefore tested if these two *T. gondii* factors contribute to delivery and persistence of mGBP1 to the *T. gondii* PV. Recruitment of mGBP1 to the vacuole of the type II PruA7 *T. gondii* strain, transgenic for the type I version of ROP16 (PruA7+ROP16I), versus the parental type II PruA7 strain, was ∼15%, and 53% respectively (Fig. 2b). A deletion of GRA15 in the same type II parental strain (PruA7ΔGRA15) led to a reduction of mGBP1 recruitment to ∼30% (Fig. 2b). Both modified type II strains exhibit a significant reduction of mGBP1 on the PV compared to wild-type parental type II. However, type I and type III strains both have a ROP16 allele that can activate STAT3/6 and an allele of GRA15 that does not activate the NFkB pathway and therefore another factor different between type I and type III must exist to explain the difference in accumulation of mGBP1 on the PVM of these strains. Recently, it was shown that type I ROP18 can directly phosphorylate two threonines in the switch 1 loop of the IRGs, thereby inactivating them. Type III strains do not express ROP18 and to determine if type I ROP18 has a role in the evasion of mGBP1 recruitment we expressed type I ROP18 in a type III strain (CEP+ROP18I) and compared its recruitment of mGBP1 to a wild-type type III strain (CEP). Recruitment of mGBP1 to the vacuole of CEP+ROP18I versus CEP, was ∼7%, and 51%, respectively, a significant difference (Fig. 2b). Thus, three polymorphic secreted *Toxoplasma* proteins determine the strain differences in recruitment of mGBP1 to the vacuole.

### GTP-binding and nucleotide-dependent multimerization are essential for re-localization of mGBP1

To investigate how activation and localization of mGBP1 are regulated by the host, and to explore which structural requirements are needed for a proper targeting to the PV of *T. gondii*, we made use of mGBP1 point mutants. GBPs consist of two structural domains. The globular N-terminal nucleotide-binding domain contains the evolutionarily conserved G1–G5 motifs of the G-domain, whereas the helical GBP C-terminal domain is more diverse. We introduced two mutations into the GTP-binding motif G1, where either arginine 48 or lysine 51 was changed to alanine. These two point mutants were based on the structure and properties of the homologous human GBP1 (hGBP1). Both mutations cause a 100–1000-fold decrease of the GTP hydrolysis rate in hGBP1 [20]. The R48A mutant is preferentially GTP-bound, since it binds the GTP analogue mant-GppNHp with higher affinity compared to the wild-type protein. The K51A mutant exhibits an up to ∼50-fold reduction in its affinity for nucleotide and is predominantly nucleotide-free and monomeric [20]. In addition, we generated a CaaX motif mutant by changing cysteine to alanine to abolish potential farnesylation of the protein. We investigated which of these altered mGBP1 versions would still be recruited to type II-containing vacuoles in IFN-γ-pretreated MEFs.

**Figure 3:**
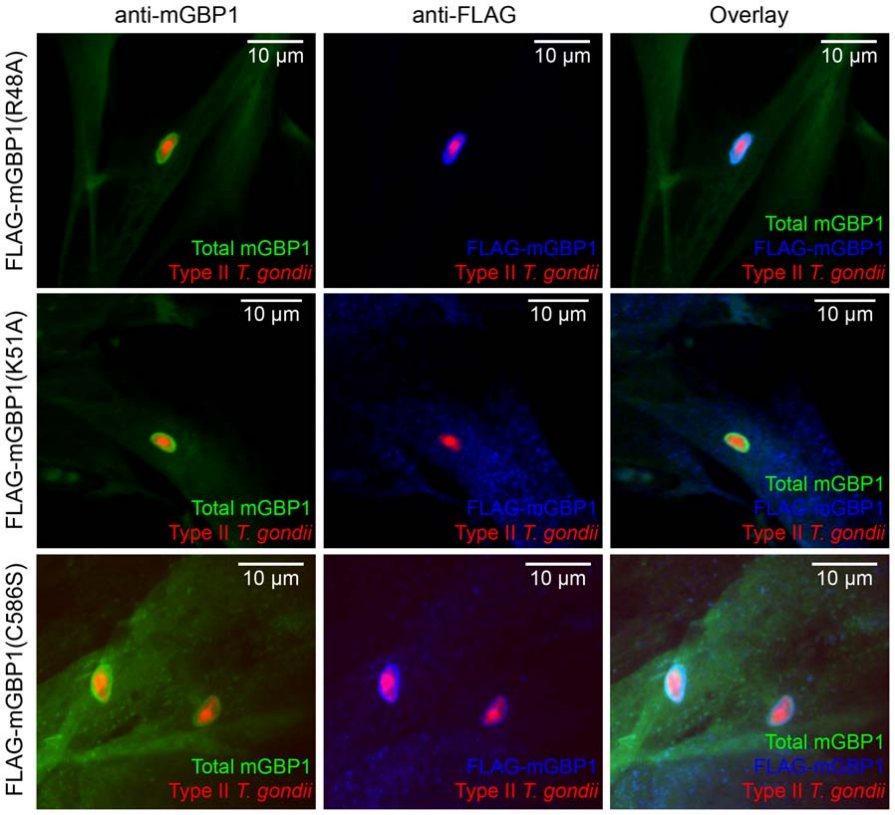
Nucleotide-dependent multimerization is required while farnesylation is dispensable for recruitment of mGBP1 to the PV. (A) Confocal pictures show MEFs that overexpress FLAG-tagged mutants of mGBP1 stimulated with 200 U/ml IFN-γ overnight and infected with mCherry-expressing type II *T. gondii* (Pru) at an MOI between 5 and 10 for 1 h. The R48A and C586S mGBP1 mutants were targeted to the PV like the wild-type counterpart. In contrast, the K51A mGBP1 mutant was unable to accumulate around the PV. Rabbit polyclonal anti-mGBP1 antiserum and a mouse monoclonal anti-FLAG antibody were used for stainings.

The R48A mutant was still targeted to the parasite-containing vacuoles in an IFN-γ-dependent manner (Fig. 3). Both intensity of accumulation and frequency of mGBP1-positive vacuoles were indistinguishable from wild-type mGBP1. In contrast, the K51A mutant was unable to target to the *T. gondii*-containing vacuoles (Fig. 3).

The abolition of farnesylation does not impact mGBP1’s ability to recognize a vacuole that contains type II *T. gondii* (Fig. 3), at least not in IFN-γ-pretreated MEFs. It has recently been shown that human GBPs can homo- and heterodimerize in tissue culture cells with the proteins always localizing to the compartment of the prenylated partner [32]. It is thus conceivable that recombinant mGBP1 is targeted to the PV by a prenylated endogenous GBP family member.

A recent study reports the critical role of the Toll-like receptor (TLR) -trafficking protein UNC93B1 in host resistance to *T. gondii*, possibly by direct exertion of its function at the PVM [33]. We therefore investigated whether TLR-mediated signaling had an impact on mGBP1 recruitment to the PVM. Since recruitment of the GTPase in both MyD88/TRIF knock-out macrophages, as well as macrophages overexpressing a mutant UNC93B1 deficient in TLR trafficking was not impaired, we concluded that signaling via TLRs prior to activation of mGBP1 was not required (Fig. S3).

Why does mGBP1 recruitment to *T. gondii* vacuoles require pre-stimulation with IFN-γ, also in cells that constitutively overexpress mGBP1? We tested if mGBP1 is modified in an IFN-γ-dependent manner in any form detectable by SDS-PAGE. When we followed the fate of metabolically labeled mGBP1 for 120 min, we failed to observe a size difference of the immunoprecipitated protein (Fig. S4 and Procedures S1). We therefore consider it less likely that mGBP1 is extensively modified in an IFN-γ-dependent manner, and favor the possible presence of an IFN-γ-induced auxiliary factor that helps recruit mGBP1 to the PV. The identity of this factor remains to be determined.

### mGBP1 acts together with mGBP2 and mGBP5 at the PV

**Figure 4:**
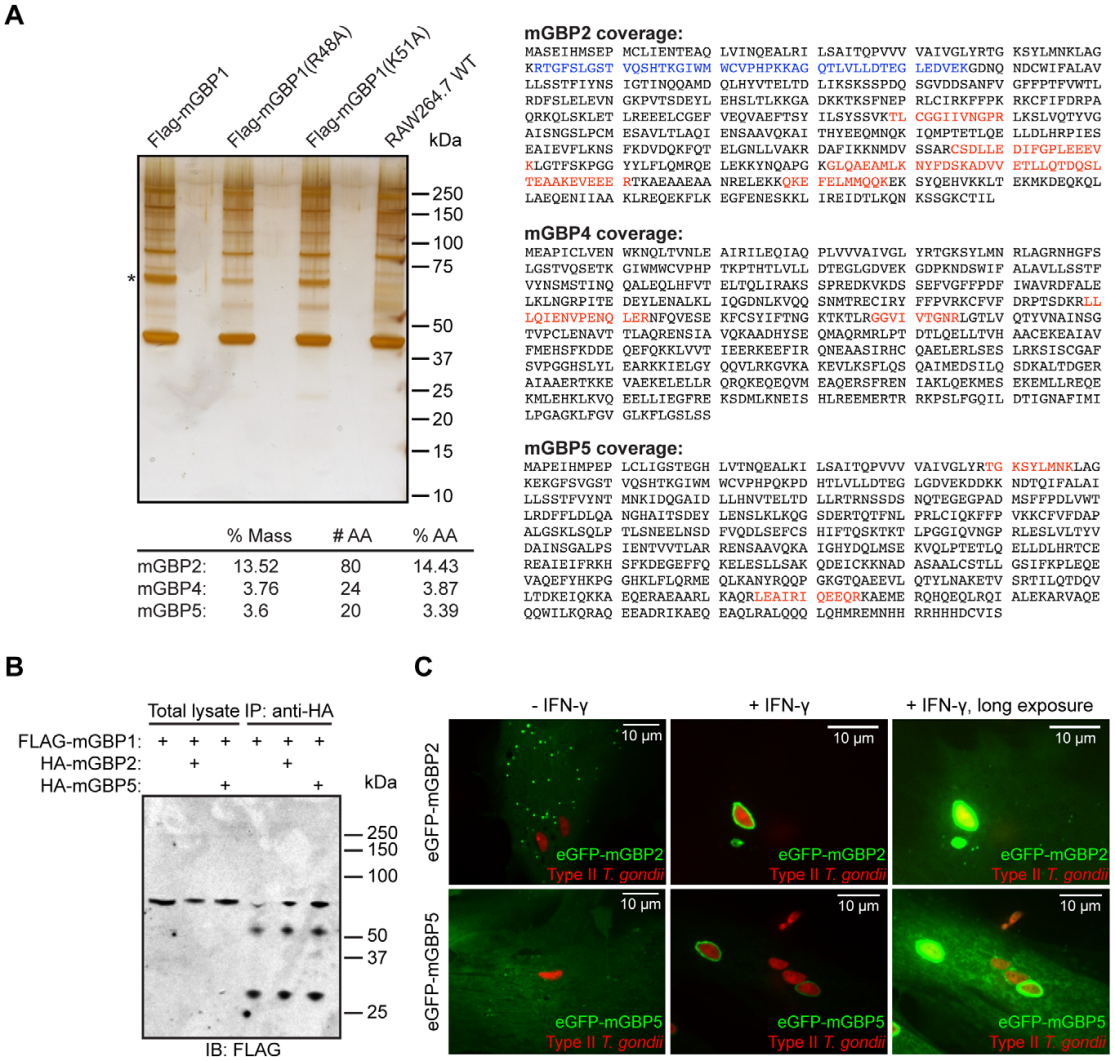
mGBP2 and mGBP5 interact with mGBP1 and accumulate on type II *T. gondii* (Pru) vacuoles. (A) mGBP2, mGBP4, and mGBP5 were identified as interactors of mGBP1 in a immunoprecipitation with subsequent MS/MS analysis (A). The left panel shows a silver-stained SDS-PAGE gel following an anti-FLAG IP from wild-type RAW264.7 cells (control lane) or RAW264.7 that overexpress wild-type or various mGBP1 mutants. The right panel shows three GBPs that were identified in the mass spectrometry analysis as binding partners of mGBP1. Wild-type RAW264.7 cells or RAW264.7 cells that overexpress FLAG-mGBP1, FLAG-mGBP1(R48A) or FLAG-mGBP1(K51A) were stimulated with 200 U/ml IFN-γ overnight and infected with type II *T. gondii* at an MOI of 5–10 for 1 h. The mGBP1 protein was recovered from all lanes except from the control lane (*). Unique peptides recovered from mGBP2, mGBP4, and mGBP5 are shown in red and peptides common with mGBP1 are shown in blue. The total number of unique amino acids (# AA), the percentage of total mass (% Mass), and the percentage of total amino acids (% AA) recovered are also shown. For mGBP4 and mGBP5, the sequence coverage was 3–4%. 14% of the mGBP2-specific amino acids were found in the gel (21% when including amino acids common to mGBP1). (B) Interaction of HA-mGBP2 and HA-mGBP5 with FLAG-mGBP1 was confirmed by co-IP with subsequent immunoblot analysis. RAW264.7 cells that overexpress either FLAG-mGBP1 and HA-mGBP2, FLAG-mGBP1 and HA-mGBP5, or FLAG-mGBP1 alone were lysed in 0.5% NP-40 and proteins were immunoprecipitated with an anti-HA antibody. Co-IP of FLAG-mGBP1 was confirmed by immunoblot with an anti-FLAG antibody. A mouse monoclonal anti-FLAG antibody (Sigma-Aldrich) and a rat monoclonal anti-HA antibody (Roche Applied Science) were used. (C) mGBP2 and mGBP5 are recruited to type II *T. gondii* vacuoles in an IFN-γ dependent manner. Confocal pictures show MEFs that overexpress either eGFP-mGBP2 or eGFP-mGBP5 pre-induced with 200 U/ml IFN-γ and infected with type II *T. gondii*. Live imaging was performed with a spinning disk confocal microscope and revealed that both mGBP2 and mGBP5 were targeted to *T. gondii* vacuoles in an IFN-γ-dependent manner.

Even though GBPs were identified as IFN-γ-regulated genes more than 25 years ago, their immunological function remains enigmatic and only two binding partners of the GBPs have been identified. NIK/HGK binds to hGBP3 and the p110 subunit of PI3K binds to mGBP2 [21], [34]. A step towards defining the molecular function of this class of GTPases in their function as anti-parasitic agents is a determination of the proteins that mGBP1 interacts with, both in the cytoplasm and on the vacuole of *T. gondii*. Furthermore, the identification of interactors would help in understanding how GBPs are activated and targeted to the PV of *T. gondii*. We generated RAW264.7 cells that stably overexpressed FLAG-tagged mGBP1 wild-type protein as well as the K51A and the R48A mutants. Cells were stimulated with IFN-γ, and whole cell lysates were used for immunoprecipitation (IP) with an anti-FLAG antibody. Co-immunoprecipitated proteins were separated by SDS-PAGE gel electrophoresis, and their identities were determined by MS/MS (Fig. 4a). We performed two independent immunoprecipitation experiments, using different lysis buffers, to confirm potential binding partners. Peptides from three other GBPs were recovered in both experiments, namely mGBP2, mGBP4 and mGBP5 (Fig. 4a). For all three proteins, peptides were only recovered in IPs of FLAG-mGBP1 and FLAG-mGBP1(R48A) but not when FLAG-mGBP1(K51A) was immunoprecipitated or in the wild-type control lane. To confirm the observed interactions, we generated RAW264.7 cells that overexpressed FLAG-tagged mGBP1 as well as either HA-tagged mGBP2 or mGBP5. IFN-γ-stimulated cells that were immunoprecipitated with anti-HA antibody, showed a FLAG-reactive band by immunoblotting at the right molecular weight of 67 kDa suggesting that mGBP1 interacts with mGBP2 and mGBP5 (Fig. 4b).

We next investigated the recruitment of mGBP2 and mGBP5 to the vacuole of type II *T. gondii* in MEFs that express either eGFP-mGBP2 or eGFP-mGBP5. Both eGFP fusion proteins of mGBP2 and mGBP5 were overexpressed in MEFs and localized to punctate structures that resembled the mGBP1 structures. We established that mGBP2 and mGBP5 were also brought to the PV in an IFN-γ-dependent manner (Fig. 4c). Localization of mGBP2 and mGBP5 to punctate structures of varying degrees was still observed when GBPs accumulated at *T. gondii* vacuole (Fig. 4c, right panel). The extent of recruitment was similar to that observed for mGBP1, and mGBP2 and mGBP5 co-localized with mGBP1 at the PV of type II *T. gondii* (Fig. S5). Furthermore, the absence of the C-terminal cysteine that would carry the farnesyl moiety does not impact the recruitment of mGBP2 and mGBP5 to the PV (Fig. S6).

Our findings suggest that various GBPs act jointly at the interface of host pathogen interaction to fight *T. gondii*. Further studies will be needed to analyze the exact regulatory mechanisms underlying the recruitment of these large GTPases.

### mGBP1 co-localizes with TGTP after infection with *T. gondii in vitro*

**Figure 5:**
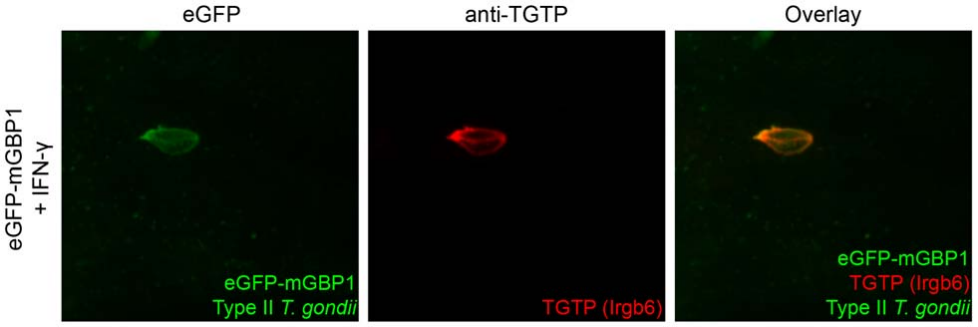
mGBP1 co-localizes with TGTP after infection with *T. gondii*. Confocal pictures show that mGBP1 co-localizes with TGTP after infection with *T. gondii* in vitro (B). MEFs that overexpress eGFP-mGBP1 were stimulated with 200 U/ml IFN-γ overnight and infected with GFP-expressing Type II *T. gondii* at a MOI between 5 and 10 for 1 h. A goat polyclonal anti-TGTP antibody was used for staining.

The IRG TGTP (Irgb6) is recruited to *T. gondii* vacuoles and is involved in GTPase-mediated disruption of the PV [7], [11]. Except for their common transcriptional regulation by IFN-γ, neither a functional link nor co-localization of GBPs with IRGs has been found. When investigated by confocal microscopy, both mGBP1 and TGTP always co-localized to identical PVs after infection with *T. gondii in vitro* (Fig. 5). Furthermore, the intensity and hence the amount of accumulated mGBP1 was comparable to the amount of TGTP detected around the PV.

This finding suggests that GBPs and IRGs act jointly at the interface of host pathogen interaction to fight *T. gondii*. Further studies will be needed to analyze the exact regulatory mechanisms underlying the recruitment of these two families of large GTPases.

## Discussion

Various IRG p47 GTPases accumulate at the PV to regulate growth and survival of *T.gondii* [6]–[8], [35]. However, many questions remain about the relevant activation pathways and mechanisms of this means of host defense. The general function of the other family of IFN-γ-inducible GTPases, the p65 GTPases, remains equally mysterious. What are the exact host and pathogen activation signals that trigger the accumulation of the GTPases on the parasitic vacuole? Why are the p47 GTPases such a large family of proteins in the mouse genome, and yet they are without an obvious functional counterpart in the human genome? Do the p65 GTPases, more equally represented in mouse and human genomes, exert a similar anti-parasitic function as do the p47 GTPases?

The immune responses elicited by the mGBP1 inducers TLR-9 and IFN-γ are important for resistance to *T. gondii* infection [29], [36]–[38], an observation consistent with a role of mGBP1 in the host response to *T. gondii*. mGBP1 localizes to discrete, vesicle-like structures in the cytoplasm and is recruited to the PV of non-virulent *T. gondii* in infected cells [29]. We confirmed these findings by demonstrating the targeting of mGBP1 to the PV after *T. gondii* infection, but only when host cells were pre-stimulated with IFN-γ. Thus, IFN-γ induces an as yet uncharacterized factor necessary for targeting of mGBP1 to the PV. Certain IRGs may be among these essential IFN-γ-dependent factors. The observation that mGBP1 was preferentially recruited to the PV of nonvirulent types II and III of *T. gondii*, and not to the virulent type I strain, as do certain IRGs, supports this hypothesis [10], [11]. An IFN-γ-dependent modification of mGBP1 would be a distinct possibility, but this suggestion lacks experimental support. Accumulation of mGBP1 at the PV was initiated already a few minutes after invasion. This demonstrates a possible role of the GBPs as part of an early cellular response to *T. gondii* infection and implies that they may be key factors of an initial cell-autonomous innate immune response. Surprisingly, we found that virulent type I parasites, for the most part, not only suppressed recruitment of mGBP1, but could also escape from a vacuole to which mGBP1 had already been recruited.

Why certain vacuoles are decorated with mGBP1, whereas others are not, is not fully understood. This applies to the family of GBPs as it does to the IRGs. However, two recent studies reported that *T. gondii* type I kinase ROP18 can directly phosphorylate the p47 IRGs IIGP1 (Irga6) and TGTP (Irgb6), which then prevents the GTPases from being recruited to the vacuole [15], [16]. Expression of ROP16 of the virulent type I *T. gondii* in a nonvirulent type II strain reduces recruitment of mGBP1 to the vacuole by more than 60%. We hypothesize that ROP16, also a kinase, may phosphorylate mGBP1 or an as yet unknown host substrate, which then impedes recruitment of mGBP1 to the PV. However, expression of ROP16 of the virulent type I and III *T. gondii* in a nonvirulent type II strain reduces virulence *in vivo* [39]. This disparity between our *in vitro* experiments and previous *in vivo* observations might be explained by two separate effects of ROP16 occurring *in vivo*. Since both type II and III parasites recruit mGBP1 to their vacuoles, it is likely that STAT3/6 activation by ROP16 of type I and III *T.gondii* and subsequent inhibition of pro-inflammatory cytokine secretion or other STAT3/6-mediated effects decrease virulence and trump interference with the GBP-mediated cell-autonomous immunity. Deletion of GRA15 in a nonvirulent type II strain also diminishes efficient recognition of the vacuole by mGBP1. GRA15 is localized to the cytoplasmic side of the vacuole and most likely exerts its effect directly on mGBP1, possibly as a stabilizing factor. GRA15 of type II parasites activates NFkB, however since recruitment of mGBP1 is observed in less than one hour and GRA15-mediated NFkB activation is only seen after four hours, we find it unlikely that a NFkB-mediated effect plays a role. Mice infected with a type II strain lacking GRA15 show a significantly increased parasite burden than mice infected with the parental type II strain [13]. Virulence of *T. gondii* in mice may therefore be partly caused by interference with the GBP-mediated host defense against the parasite. Further studies are needed to analyze the exact molecular mechanism of the recognition function of these two *T. gondii* factors. We also found that ROP18 plays a significant role in the evasion of GBP1 recruitment as a type III strain expressing type I ROP18 (CEP+ROP18I) is able to evade recruitment. In fact, the recruitment of III+ROP18I is similar to the recruitment of type I strain suggesting that ROP18 is the only factor mediating the difference in recruitment between type I and type III strains. How ROP18 mediates its effects on mGBP1 recruitment remains to be investigated. It is possible that ROP18 directly phosphorylates mGBP1 and thereby affects its recruitment or the effect could be indirect. For example if recruitment of the IRGs is necessary for the GBPs to be recruited to the vacuole then ROP18 inhibition of IRG recruitment could also affect GBP recruitment.

Three point mutants of mGBP1, R48A, K51A, and C586S, were used to investigate how activation and localization of mGBP1 are regulated by the host. mGBP1(R48A) is still recruited to the vacuole, despite having a more than 100-fold reduced rate of GTP hydrolysis. This mutant is probably GTP-bound, implying that the nucleotide-bound state and nucleotide-dependent multimerization are important, while GTP hydrolysis is dispensable for targeting of mGBP1 to the PV membrane. The inability of the nucleotide-empty and monomeric K51A mutant to re-localize to the PV supports this hypothesis. In contrast to the properties reported for human GBP1 [22], we find that farnesylation is dispensable for a correct subcellular localization of the GBPs. However, since GBPs can form dimers and experiments were performed in wild-type cells, the farnesylation mutants may still dimerize with wild-type, farnesylated proteins to explain their recruitment to the PV. Considering that only 15% of mGBP1 is farnesylated [40], other posttranslational modifications might be present and be important for proper targeting of the GTPase. However, we did not detect obvious signs of modification of GBPs in pulse-chase labeling experiments upon IFN-γ stimulation of cells. Also, activation of mGBP1 did not require prior engagement of TLRs.

We did not observe localization of mGBP1 to the PV of *T. cruzi* or to phagosomes containing zymosan A. The GBPs are therefore most likely a specific response to subset of intracellular pathogens, including *T. gondii*, rather than serve as general regulators of phagocytosis and phagosomal degradation. The *T. gondii*-specific re-localization of mGBP1, mGBP2, and mGBP5 suggests that certain GBPs act in concert to fulfill their functions after induction by IFN-γ. However, variations in the C-terminal parts of the GBPs suggest that each GBP has a specific role in this complex to mediate resistance against certain intracellular pathogens.

IP data confirmed interactions between members of the mouse GBP family, as reported also for the IRGs [41] and human GBPs [32]. mGBP1 interacts with mGBP2, mGBP5, and possibly with mGBP4. Both mGBP2 and mGBP5 are brought to the PV of *T. gondii* in a manner similar to mGBP1, with farnesylation dispensable for targeting to the PV. The recruitment of mGBP5 to the *T. gondii* type II PV is a finding at variance with published negative results [29], which could be the result of the antibody employed.

The highly conserved p65 GBPs join the p47 IRGs as elements of cell-autonomous immunity against certain intracellular pathogens. Several GBPs are re-localized to the PV of *T. gondii* minutes after invasion to counteract the parasite. Virulent strains of *T. gondii* have evolved mechanisms to evade the recognition by the host cell and interfere with activation of the GBPs through injection of virulence factors like ROP16 and ROP18. Further investigations along the lines of our findings are needed to clearly define the role of all GBP family members, their regulation and interaction with the p47 IRGs specifically. Identification of the first parasitic factors that are able to control cellular localization of the GBPs, as well as knowing that the family members form complexes with each other provides a strong starting point for these studies.

## Materials and Methods

### Immunochemical Reagents

The following immunological reagents were used for immunoblotting, immunoprecipitation and immunofluorescence: mouse monoclonal anti-FLAG antibody (clone M2, Sigma-Aldrich), rat monoclonal anti-HA antibody (clone 3F10, Roche Applied Science), goat polyclonal anti-*T. gondii* antibody (Abcam), and rabbit polyclonal anti-mGBP1 antiserum (raised against recombinant full-length mGBP1). Mouse IFN-γ was purchased from PeproTech Inc.

### Cloning

Full-length cDNA clones from Open Biosystems encoding mGBP1 (BC108990), mGBP2 (BC011336), and mGBP5 (BC058555) were cloned into pMSCV vectors for retroviral expression. N-terminal FLAG- and HA-epitope tags were introduced by primers. Site-directed mutagenesis was performed using the QuikChange II Site-Directed Mutagenesis Kit (Agilent Technologies Inc.) to introduce the R48A and K51A mutations into mGBP1. The C586S and C587S mutations were introduced to the coding sequences by primers.

### Primer sequences

See Table S1.

### Cell culture and parasite strains

Human foreskin fibroblasts (HFFs), RAW264.7 cells, LLcMK2 cells, and mouse immortalized macrophages were cultivated in Dulbecco’s modified Eagle medium (DMEM) containing 10% fetal calf serum (FCS), 2 mM L-glutamine, and penicillin-streptomycin. 129/SvEv × C57BL/6 mouse embryonic fibroblasts (MEFs) were cultivated in DMEM supplemented with 15% FCS, 2 mM L-glutamine, 0.1 mM non-essential amino acids, 115 µM β-mercaptoethanol and penicillin-streptomycin. Immortalized macrophages from wild-type and MyD88/TRIF double-knockout mice were a kind gift from Douglas Golenbock (University of Massachusetts Medical School, Worcester, MA). Immortalization of macrophages has been described earlier [42]. 3d mice that overexpress a mutant Unc93B1 were a kind gift of Dr. Bruce Beutler (The Scripps Research Institute, La Jolla, CA) [43]. *T. gondii* strains CEP, Pru, PruA7, and RH were propagated in HFF monolayers as described previously [44]. All four strains were genetically modified to drive cytosolic expression of a fluorescent protein. The Pru Δhxgprt fLUCGFP strain (PruA7) [45] was used to construct PruA7 *GRA15* gene deletion and *ROP16* transgenic parasite strains. The generation of PruA7ΔGRA15 has been described recently [13]. To generate PruA7+ROP16I parasites and CEP+ROP18I parasites, a hemagglutinin-tagged ROP16I or ROP18I expression construct containing 2 kb of upstream genomic region flanking the start codon of the type I ROP16/ROP18 gene was cloned and shuttled by LR recombination to a Gateway (Invitrogen) compatible destination vector pTKO-att [13], which flanks inserts with the Toxoplasma GRA2 3′ UTR and contains the selectable hxgprt gene. To generate PruA7+ROP16I and CEP+ROP18I parasites, the linearized ROP16I/ROP18I pTKO-att expression construct was transfected by electroporation into PruA7 or CEPhpt^−^ parasites. Stable integrants were selected with mycophenolic acid/xanthine and cloned by limiting dilution [46]. Successful integration, expression in the rhoptries, and functionality of ROP16I/ROP18I was confirmed by immunofluorescence staining for the HA tag and nuclear translocation of phosphorylated STAT6 in infected HFFs (PruA7+ROP16I) or enhancement of in vivo virulence (CEP+ROP18I). *T. cruzi*, Tulahuén strain, was maintained in LLcMK2 cells.

### Co-Immunoprecipitation

IFN-γ-induced RAW264.7 cells were lysed for 30 min at 4°C in 0.5% NP-40 lysis buffer (25 mM Tris HCl pH 7.4, 150 mM NaCl, 5 mM MgCl2, cOmplete EDTA-free Protease Inhibitor Cocktail (Roche) and 0.5 mM GTP) and nuclear fraction was removed by centrifugation. Supernatants were pre-cleared for 3 h at 4°C with Protein G beads and subsequently incubated for 2 h at 4°C with a rat monoclonal anti-HA antibody (Roche Applied Science) and Protein G beads. Bound proteins were eluted from the washed beads by boiling at 95°C in Laemmli buffer and subjected to SDS–PAGE and IB.

### Large-scale immunoprecipitation and mass spectrometry

RAW264.7 cells were stimulated with 200 U/ml IFN-γ overnight, infected with *T. gondii* Pru for 1 h at an MOI of 5–10. Proteins were co-immunoprecipitated with a mouse monoclonal anti-FLAG antibody (Sigma-Aldrich) as described above. Bound proteins were eluted from the washed beads with 200 µg/ml FLAG peptide and subjected to SDS–PAGE and silver staining. For mass spectrometry analysis, single bands were excised from each lane of a silver stained SDS-PAGE gel encompassing the entire molecular weight range. Trypsin digested extracts were analyzed by reversed phase HPLC and a ThermoFisher LTQ linear ion trap mass spectrometer. Peptides were identified from the MS data using SEQUEST algorithms44 that searched a species-specific database generated from NCBI's non-redundant (nr.fasta) database.

### Immunofluorescence and live-cell imaging

Immunofluorescence was performed in Lab-Tek™ II - CC2™ Chamber Slide™ System slides (Nalge Nunc International) and cells were grown in standard growth media at 37°C and 5% CO2. Cells were stimulated with 200 U/ml IFN-γ overnight and infected with *T. gondii* at an MOI of 5–10. After the indicated time, cells were fixed in 2% PFA for 30 min at RT. Washed cells were permeabilized in 0.1% Triton X-100/PBS for 10 min. Non-fluorescent proteins were visualized by antibody staining. Samples were imaged with a spinning disk confocal microscope. The frequency of mGBP1-positive vacuoles was determined by counting at least 100 *T. gondii*-containing PVs. A vacuole was counted as mGBP1-positive when a distinct and clear circular accumulation of mGBP1 around the PV was visible. Live-cell imaging was performed in Lab-Tek™ II Chambered Coverglass slides (Nalge Nunc International). Cells were treated as described above, but not fixed. Instead, cells were imaged live at 37°C and 5% CO2 using a spinning disk confocal microscope.

## Supporting Information

Figure S1 **mGBP1 is localized to punctate structures in the cytoplasm.** MEFs that overexpress FLAG-mGBP1 or eGFP-mGBP1 were stimulated with 200 U/ml IFN-γ overnight and stained with an anti-mGBP1 antiserum to detect the endogenous and tagged protein. Following IFN-γ stimulation, the mGBP1 protein was localized to punctate structures within the cytoplasm. Most of the punctae stained by anti-mGBP1 were also detected with the anti-FLAG antibody, indicating co-localization of tagged mGBP1 with the endogenous mGBP1 protein pool. Similar results were obtained for the eGFP fusion protein of mGBP1. Localization patterns for both constructs and the endogenous mGBP1 were similar in cells without IFN-γ pre-stimulation (data not shown). Rabbit polyclonal anti-mGBP1 antiserum and a mouse monoclonal anti-FLAG antibody (Sigma-Aldrich) were used for stainings. Pictures were taken with a spinning disk confocal microscope.
(TIF)

Figure S2 **mGBP1 is not recruited to other compartments within the host cell.** (A) mGBP1 was not recruited to zymosan-containing phagosomes. Mouse immortalized macrophages were stimulated with 200 U/ml IFN-γ overnight and incubated with zymosan-Alexa 647 for 1 h to allow phagocytosis of labeled zymosan A. Rabbit polyclonal anti-mGBP1 antiserum was used for staining. Arrowheads point to diffuse accumulation of mGBP1 in the vicinity of the phagosome. (B) mGBP1 is not recruited to the PV of *T. cruzi*. MEFs were stimulated with 200 U/ml IFN-γ overnight and infected with *Trypanosoma cruzi* for 1 h. Rabbit polyclonal anti-mGBP1 antiserum and DAPI were used for stainings. *T. cruzi* was visualized by DAPI stain that also labeled nuclear DNA of the host cell. Arrows point to *T. cruzi.* Pictures were taken with a spinning disk confocal microscope.
(TIF)

Figure S3 **mGBP1 recruitment to** ***T. gondii*** **is independent from TLR signaling and Unc93B1.** Confocal pictures show MEFs from 3d mice that overexpress mutant Unc93B1 and mouse immortalized macrophages from MyD88/TRIF double-knockout mice that were infected with type II *T. gondii* (Pru) and stained for mGBP1. MEFs from 3d mice and mouse immortalized macrophages from MyD88/TRIF double-knockout mice were stimulated with 200 U/ml IFN-γ overnight and infected with mCherry expressing type II *T. gondii* at an MOI of 5 to 10 for 1 h. Rabbit polyclonal anti-mGBP1 antiserum was used to detect endogenous mGBP1. Neither expression of dominant negative Unc93B1 or abolition of MyD88/TRIF prevents recruitment of mGBP1 to the PV.
(TIF)

Figure S4 **mGBP1 is not extensively modified upon stimulation with IFN-γ.** SDS-PAGE gel shows whole cell lysates of radioactively labeled RAW264.7 after IP with anti-FLAG and anti-mGBP1 antibodies. RAW264.7 cells were stimulated with 200 U/ml IFN-γ overnight, starved for 1 h, metabolically labeled with [^35^S] for 10 min, and chased for the indicated time. At the end of each time point, cells were lysed in 0.5% NP-40 lysis buffer and IPs and re-IPs were performed with a rabbit polyclonal anti-mGBP1 antiserum and a mouse monoclonal anti-FLAG antibody (Sigma-Aldrich). Immunoprecipitated samples were separated on a 10% SDS-PAGE gel, dried on a Whatman paper and analyzed by fluorography. FLAG-tagged mGBP1 was visible at 68 kDa (*), and endogenous mGBP1 was detected at 66 kDa (**). An IFN-γ-independent, unidentified protein was co-immunoprecipitated by both antibodies (***).
(TIF)

Figure S5 **mGBP2/5 co-localize with mGBP1 at the PV of type II** ***T. gondii*** **upon IFN-γ pre-stimulation.** Pictures were taken with a spinning disk confocal microscope. MEFs that overexpress either FLAG-mGBP1/HA-mGBP2 or FLAG-mGBP1/HA-mGBP2 were stimulated with 200 U/ml IFN-γ overnight and infected with mCherry-expressing type II *T. gondii* (Pru) at an MOI between 5 and 10 for 1 h. A mouse monoclonal anti-FLAG antibody (Sigma-Aldrich) and a rat monoclonal anti-HA antibody (Roche Applied Science) were used to stain for FLAG-mGBP1, HA-mGBP2, and HA-mGBP5. HA-mGBP2 and HA-mGBP5 co-localized with mGBP1 at the PV of type II *T. gondii* upon stimulation with IFN-γ.
(TIF)

Figure S6 **Farnesylation of mGBP2 and mGBP5 is dispensable for targeting to type II** ***T. gondii*** **vacuoles.** Confocal pictures show MEFs that overexpress either HA-mGBP2(C586S) or HA-mGBP5(C587S) infected with type II *T. gondii* (Pru). MEFs that overexpress HA-mGBP2(C586S) or HA-mGBP5(C587S) were stimulated with 200 U/ml IFN-γ overnight and infected with type II *T. gondii* for 1 h. Cells were stained with a rat monoclonal anti-HA antibody (Roche Applied Science). mGBP2 and mGBP5 were able to localize to PVs without any modification by prenyltransferases.
(TIF)

Table S1 **Primer sequences.** Primer sequences for cloning primers and quikchange primers are listed. In addition to the sequences, the corresponding restriction enzymes used for digestion, the introduced mutations, the binding sites or the product lengths are shown.
(TIF)

Procedures S1 **Supplemental Experimental Procedures.**
(DOC)

Video S1 MEFs that overexpress eGFP-mGBP1 were stimulated with 200 U/ml IFN-γ overnight and infected with mCherry-expressing type I *T. gondii* at an MOI between 5 and 10. Video was started 40 min post-infection, and images were collected every 20 seconds. This video shows 6 frames per second.
(MP4)

Video S2 MEFs that overexpress eGFP-mGBP1 were stimulated with 200 U/ml IFN-γ overnight and infected with mCherry-expressing type I *T. gondii* at an MOI between 5 and 10. Video was started 90 min post-infection, and images were collected every 45 seconds. This video shows 3 frames per second.
(MP4)

Video S3 MEFs that overexpress eGFP-mGBP1 were stimulated with 200 U/ml IFN-γ overnight and infected with mCherry-expressing type I *T. gondii* at an MOI between 5 and 10. Video was started 180 min post-infection, and images were collected every 30 seconds. This video shows 15 frames per second.
(MP4)
