# Supplementary figures and images for: Determinants of GBP Recruitment to Toxoplasma gondii Vacuoles and the Parasitic Factors That Control It
Source: PLoS One. 2011 Sep 8;6(9):e24434. doi: 10.1371/journal.pone.0024434 (PMC3169597; doi:10.1371/journal.pone.0024434)

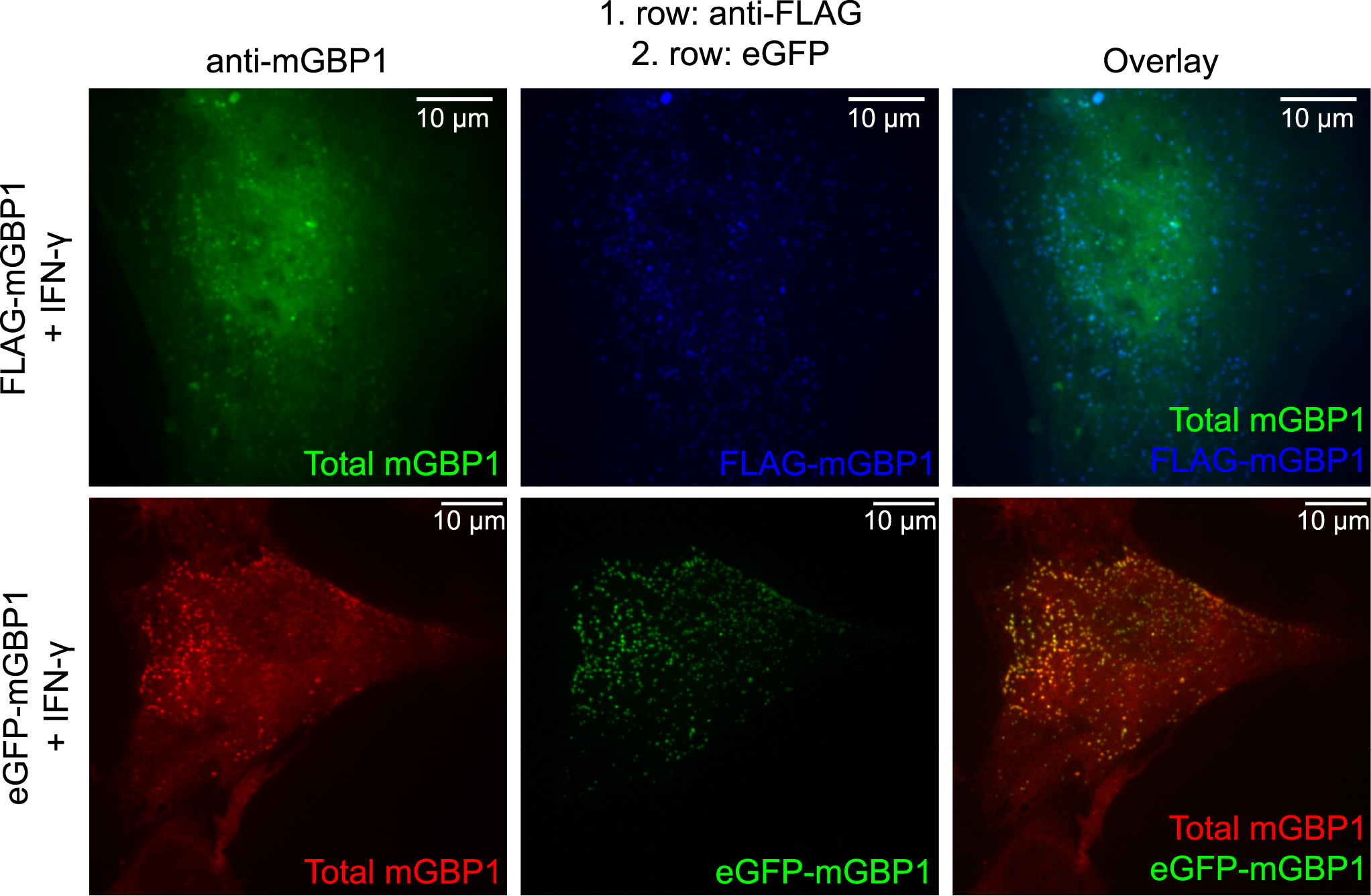

Supplement: Figure S1 — mGBP1 is localized to punctate structures in the cytoplasm. MEFs that overexpress FLAG-mGBP1 or eGFP-mGBP1 were stimulated with 200 U/ml IFN-γ overnight and stained with an anti-mGBP1 antiserum to detect the endogenous and tagged protein. Following IFN-γ stimulation, the mGBP1 protein was localized to punctate structures within the cytoplasm. Most of the punctae stained by anti-mGBP1 were also detected with the anti-FLAG antibody, indicating co-localization of tagged mGBP1 with the endogenous mGBP1 protein pool. Similar results were obtained for the eGFP fusion protein of mGBP1. Localization patterns for both constructs and the endogenous mGBP1 were similar in cells without IFN-γ pre-stimulation (data not shown). Rabbit polyclonal anti-mGBP1 antiserum and a mouse monoclonal anti-FLAG antibody (Sigma-Aldrich) were used for stainings. Pictures were taken with a spinning disk confocal microscope. (TIF) [file pone.0024434.s001.tif]

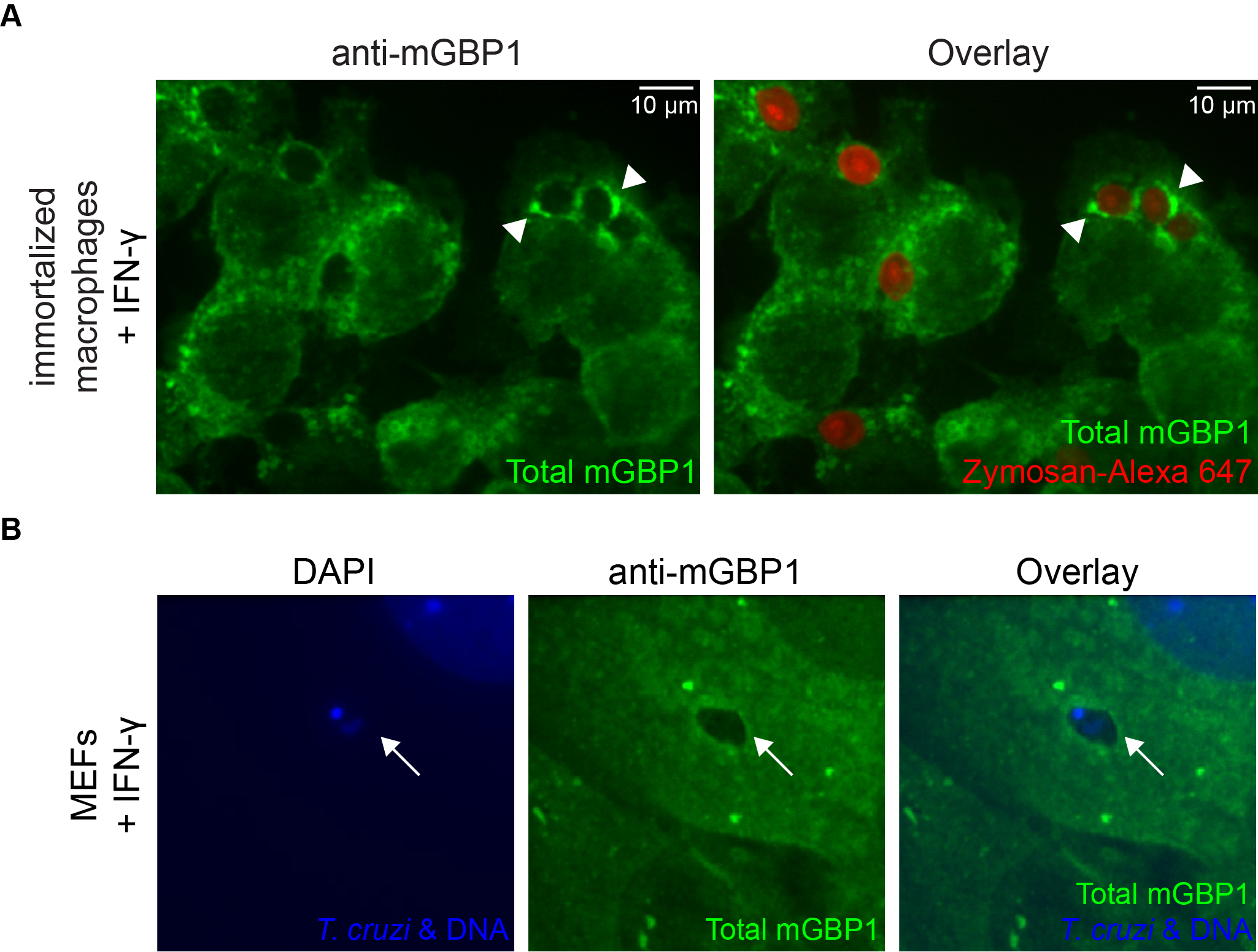

Supplement: Figure S2 — mGBP1 is not recruited to other compartments within the host cell. (A) mGBP1 was not recruited to zymosan-containing phagosomes. Mouse immortalized macrophages were stimulated with 200 U/ml IFN-γ overnight and incubated with zymosan-Alexa 647 for 1 h to allow phagocytosis of labeled zymosan A. Rabbit polyclonal anti-mGBP1 antiserum was used for staining. Arrowheads point to diffuse accumulation of mGBP1 in the vicinity of the phagosome. (B) mGBP1 is not recruited to the PV of T. cruzi. MEFs were stimulated with 200 U/ml IFN-γ overnight and infected with Trypanosoma cruzi for 1 h. Rabbit polyclonal anti-mGBP1 antiserum and DAPI were used for stainings. T. cruzi was visualized by DAPI stain that also labeled nuclear DNA of the host cell. Arrows point to T. cruzi. Pictures were taken with a spinning disk confocal microscope. (TIF) [file pone.0024434.s002.tif]

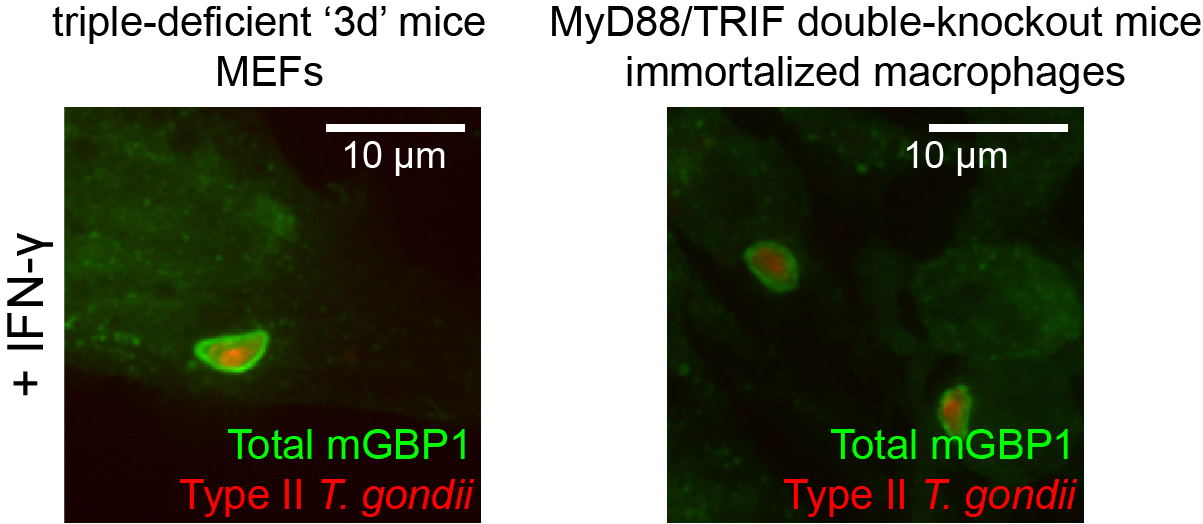

Supplement: Figure S3 — mGBP1 recruitment to T. gondii is independent from TLR signaling and Unc93B1. Confocal pictures show MEFs from 3d mice that overexpress mutant Unc93B1 and mouse immortalized macrophages from MyD88/TRIF double-knockout mice that were infected with type II T. gondii (Pru) and stained for mGBP1. MEFs from 3d mice and mouse immortalized macrophages from MyD88/TRIF double-knockout mice were stimulated with 200 U/ml IFN-γ overnight and infected with mCherry expressing type II T. gondii at an MOI of 5 to 10 for 1 h. Rabbit polyclonal anti-mGBP1 antiserum was used to detect endogenous mGBP1. Neither expression of dominant negative Unc93B1 or abolition of MyD88/TRIF prevents recruitment of mGBP1 to the PV. (TIF) [file pone.0024434.s003.tif]

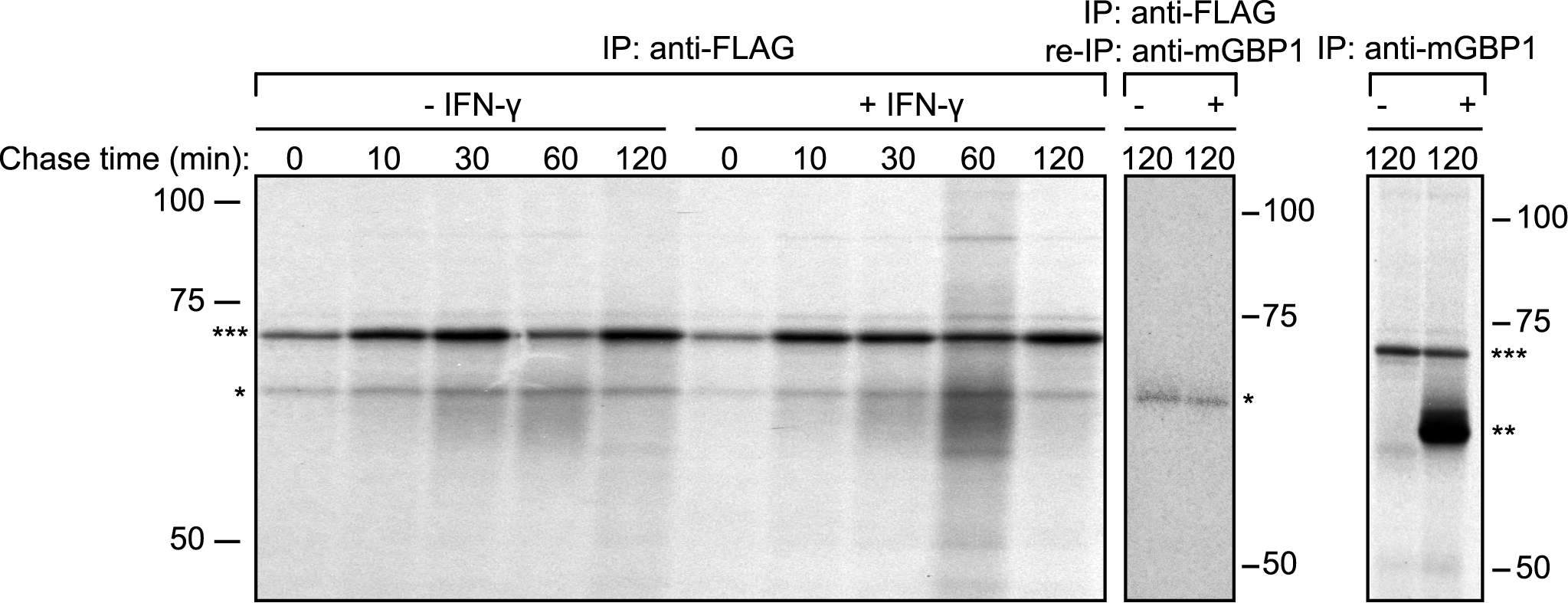

Supplement: Figure S4 — mGBP1 is not extensively modified upon stimulation with IFN-γ. SDS-PAGE gel shows whole cell lysates of radioactively labeled RAW264.7 after IP with anti-FLAG and anti-mGBP1 antibodies. RAW264.7 cells were stimulated with 200 U/ml IFN-γ overnight, starved for 1 h, metabolically labeled with [35S] for 10 min, and chased for the indicated time. At the end of each time point, cells were lysed in 0.5% NP-40 lysis buffer and IPs and re-IPs were performed with a rabbit polyclonal anti-mGBP1 antiserum and a mouse monoclonal anti-FLAG antibody (Sigma-Aldrich). Immunoprecipitated samples were separated on a 10% SDS-PAGE gel, dried on a Whatman paper and analyzed by fluorography. FLAG-tagged mGBP1 was visible at 68 kDa (*), and endogenous mGBP1 was detected at 66 kDa (**). An IFN-γ-independent, unidentified protein was co-immunoprecipitated by both antibodies (***). (TIF) [file pone.0024434.s004.tif]

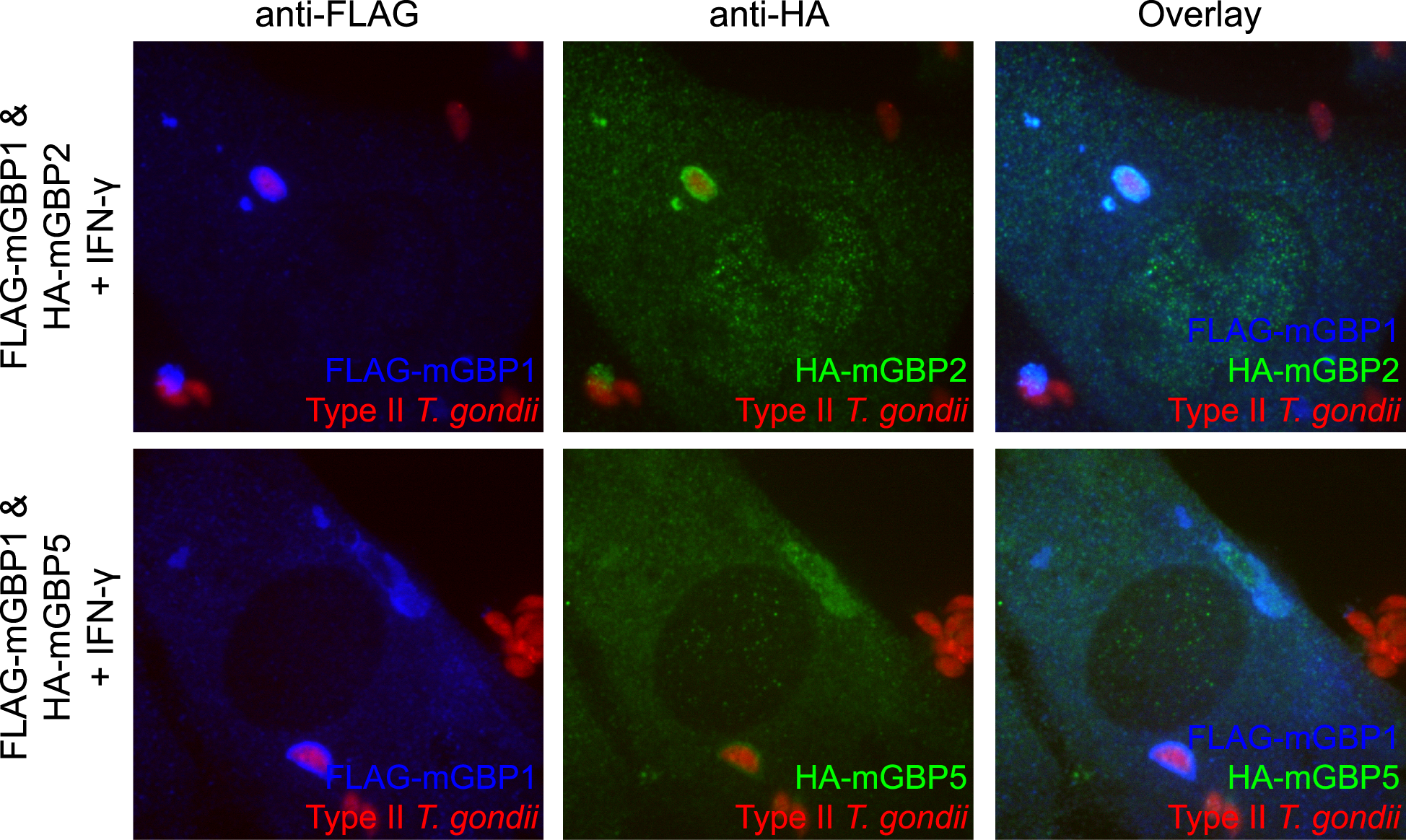

Supplement: Figure S5 — mGBP2/5 co-localize with mGBP1 at the PV of type II T. gondii upon IFN-γ pre-stimulation. Pictures were taken with a spinning disk confocal microscope. MEFs that overexpress either FLAG-mGBP1/HA-mGBP2 or FLAG-mGBP1/HA-mGBP2 were stimulated with 200 U/ml IFN-γ overnight and infected with mCherry-expressing type II T. gondii (Pru) at an MOI between 5 and 10 for 1 h. A mouse monoclonal anti-FLAG antibody (Sigma-Aldrich) and a rat monoclonal anti-HA antibody (Roche Applied Science) were used to stain for FLAG-mGBP1, HA-mGBP2, and HA-mGBP5. HA-mGBP2 and HA-mGBP5 co-localized with mGBP1 at the PV of type II T. gondii upon stimulation with IFN-γ. (TIF) [file pone.0024434.s005.tif]

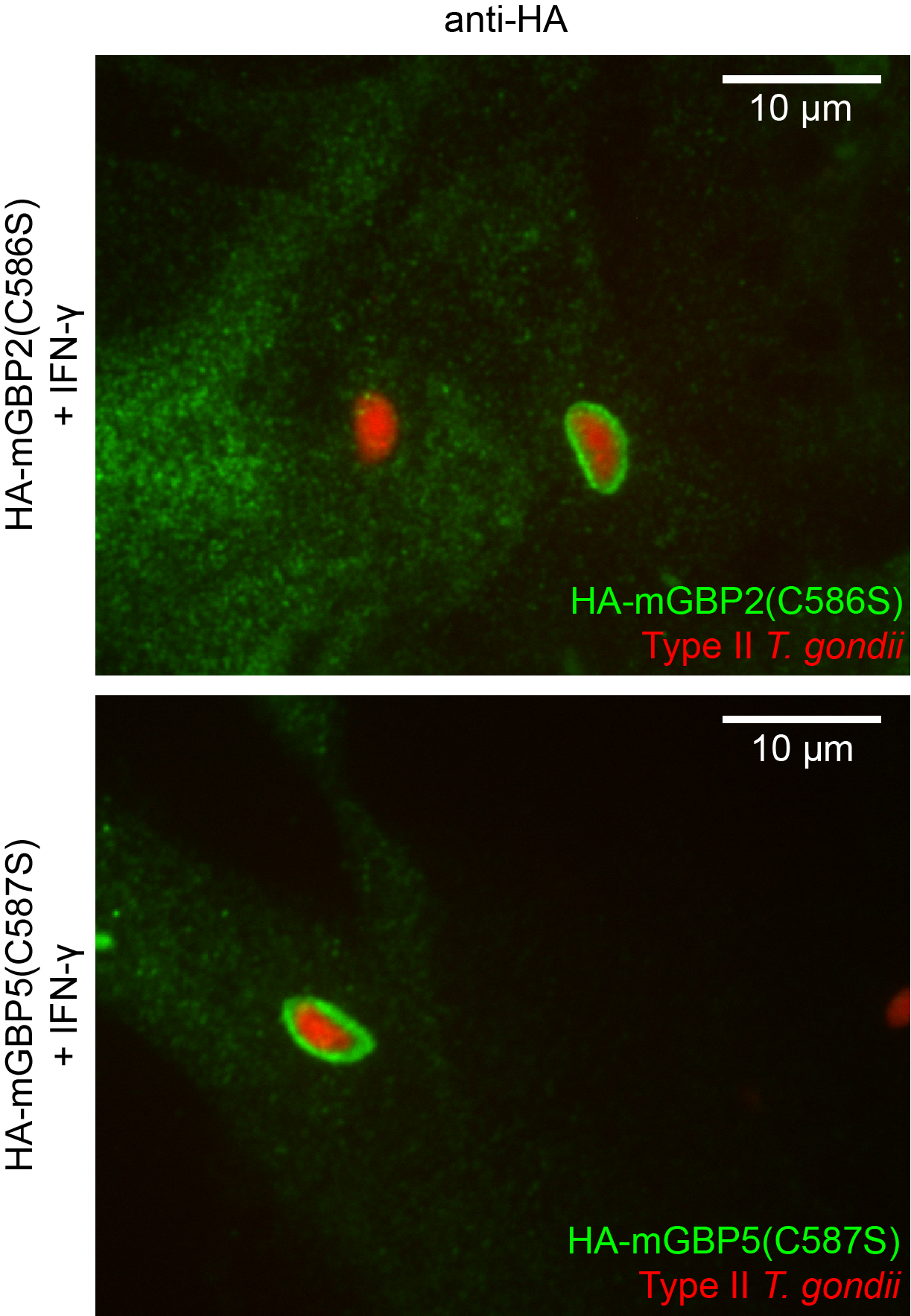

Supplement: Figure S6 — Farnesylation of mGBP2 and mGBP5 is dispensable for targeting to type II T. gondii vacuoles. Confocal pictures show MEFs that overexpress either HA-mGBP2(C586S) or HA-mGBP5(C587S) infected with type II T. gondii (Pru). MEFs that overexpress HA-mGBP2(C586S) or HA-mGBP5(C587S) were stimulated with 200 U/ml IFN-γ overnight and infected with type II T. gondii for 1 h. Cells were stained with a rat monoclonal anti-HA antibody (Roche Applied Science). mGBP2 and mGBP5 were able to localize to PVs without any modification by prenyltransferases. (TIF) [file pone.0024434.s006.tif]

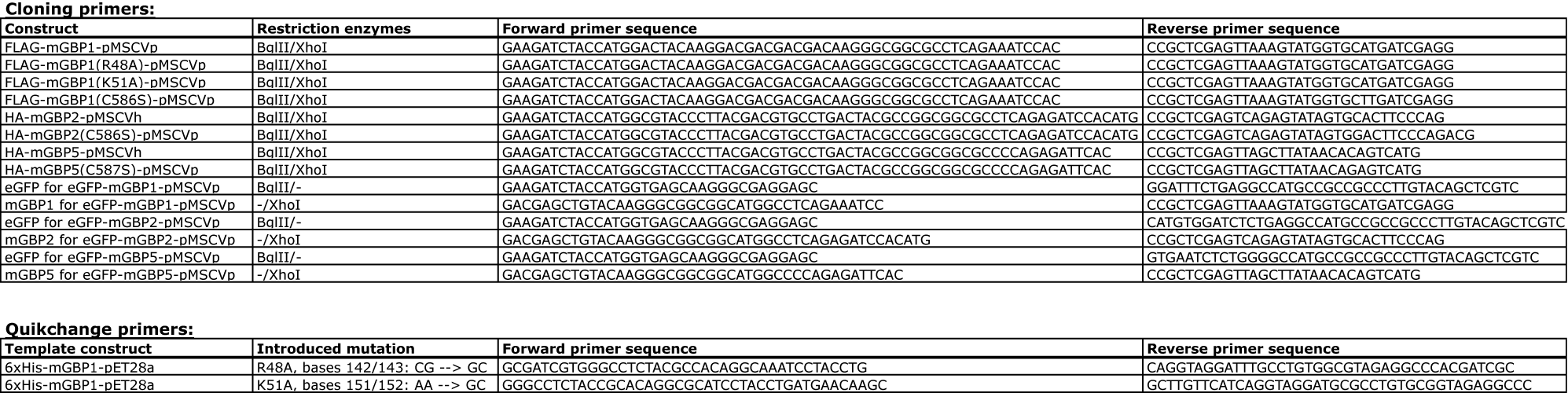

Supplement: Table S1 — Primer sequences. Primer sequences for cloning primers and quikchange primers are listed. In addition to the sequences, the corresponding restriction enzymes used for digestion, the introduced mutations, the binding sites or the product lengths are shown. (TIF) [file pone.0024434.s007.tif]
