# Supplementary material for: Determinants of GBP Recruitment to Toxoplasma gondii Vacuoles and the Parasitic Factors That Control It
Source: PLoS One. 2011 Sep 8;6(9):e24434. doi: 10.1371/journal.pone.0024434 (PMC3169597; doi:10.1371/journal.pone.0024434)
Supplement: Procedures S1 — Supplemental Experimental Procedures. (DOC) [file pone.0024434.s008.doc]

**Supplemental Experimental Procedures**

Phagocytosis assay

Zymosan A from *Saccharomyces cerevisiae* (Sigma-Aldrich) was labeled with Alexa Fluor® 647 carboxylic acid, succinimidyl ester (Invitrogen). To investigate recruitment of mGBP1 to phagosomes, zymosan solution was added to mouse immortalized macrophages for 1 h to allow phagocytosis. Cells were washed twice with PBS, fixed, and stained for mGBP1 as described above.

Pulse-chase

RAW264.7 cells were starved in methionine- and cysteine-free medium for 1.5 h and subsequently pulse labeled for 10 min in 250 μCi/ml of [^35^S]methionine and [^35^S]cysteine. At the end of each time point, cells were rinsed twice with PBS and lysed in NP-40 lysis buffer (25 mM Tris HCl pH 7.4, 150 mM NaCl, 5 mM MgCl2, cOmplete EDTA-free Protease Inhibitor Cocktail (Roche) and 0.5 mM GTP) for 1 h. Samples were equilibrated by scintillation counting (LS 6500 Liquid Scintillation Counter, Beckman) and used for IP and Re-IP. Immunoprecipitated proteins were separated by SDS-PAGE, and metabolically labeled proteins were detected by fluorography [47]. Gels were exposed to Kodak™ BioMax® MR films under safelight conditions and kept at -80°C for several days before films were developed.
